# Pyrite-induced uv-photocatalytic abiotic nitrogen fixation: implications for early atmospheres and Life

**DOI:** 10.1038/s41598-019-51784-8

**Published:** 2019-10-25

**Authors:** E. Mateo-Marti, S. Galvez-Martinez, C. Gil-Lozano, María-Paz Zorzano

**Affiliations:** 1 Centro de Astrobiología (CSIC-INTA), Ctra. Ajalvir, Km. 4, 28850-Torrejón de Ardoz, Madrid, Spain; 2 Department of Computer Science, Electrical and Space Engineering, Luleå Universit of Technology, 97187 Luleå, Sweden

**Keywords:** Planetary science, Astrobiology

## Abstract

The molecular form of nitrogen, N_2_, is universally available but is biochemically inaccessible for life due to the strength of its triple bond. Prior to the emergence of life, there must have been an abiotic process that could fix nitrogen in a biochemically usable form. The UV photo-catalytic effects of minerals such as pyrite on nitrogen fixation have to date been overlooked. Here we show experimentally, using X-ray photoemission and infrared spectroscopies that, under a standard earth atmosphere containing nitrogen and water vapour at Earth or Martian pressures, nitrogen is fixed to pyrite as ammonium iron sulfate after merely two hours of exposure to 2,3 W/m 2 of ultraviolet irradiance in the 200–400 nm range. Our experiments show that this process exists also in the absence of UV, although about 50 times slower. The experiments also show that carbonates species are fixed on pyrite surface.

## Introduction

Nitrogen is an essential element for life, as we know it. It is included in all enzymes and genes. The atmosphere of Earth has about 80% of molecular nitrogen N_2_. Nitrogen fixation on Earth is nowadays predominantly biological and occurs by conversion of N_2_ to ammonia via enzyme-catalysed reactions. N_2_ is exceptionally inert because of its triple bond and will thus not react easily with other chemicals as each one of the bonds requires 9.79 eV to be broken.

A prerequisite for the origin and evolution of life on Earth, or in any other potentially habitable planet, is the existence of some abiotic process that provides a source of fixed nitrogen, in a form that is biochemically usable^1^. Because of the strong binding of nitrogen, some previously described natural abiotic nitrogen fixation mechanisms which have been postulated on Earth were very energetic, examples of them include lightening, volcanism^2^ and meteoric impact on ancient oceans^3^. It has also been argued that coronal mass ejection events from the young Sun, produced very energetic particles that initiated reactions converting molecular nitrogen, methane, and carbon dioxide into HCN, NO, and N_2_O in the early Earth^4^. Nitrogen is also a component of the Martian atmosphere, and it has been found on the regolith of Gale crater, in the form of nitrates^5^. Recent experimental work has shown that nitrogen may have been fixed on Mars by bolide impacts in CO_2_‐N_2_ atmospheres as nitrogen oxide NO, with a high fixation rate when this is done in the presence of hydrogen (H_2_)^6^. Other energy sources such as cosmic rays, corona and lightning discharges from thunderstorms, and heat from volcanoes have also been considered as plausible processes with a minor role in nitrogen fixation on Mars^7^. The industrial ammonia synthesis by the Haber-Bosch process is also very energy-intensive. It uses hydrogen and an iron-based metal catalyst, under high temperature (400–500 °C) and pressures (150–250 atm). Numerous studies have focused on finding ways to reduce the energy requirements for the synthesis of ammonia NH_3_ from N_2_ using new heterogeneous catalysers^8^.

Nitrogen fixation requires breaking the strong bonds that hold nitrogen atoms in pairs in gaseous phase in the atmosphere and using the resulting nitrogen atoms to create molecules such as ammonia, which is the building block of many complex organics, including proteins, DNA, RNA etc. In the biological cycle of nitrogen, nitrogen fixation occurs under ambient conditions and is done by a small but diverse group of organisms that contain a metalloenzyme called nitrogenase which catalyses the conversion of N_2_ into NH_3_. Interestingly, the active centre of nitrogenase is in essence an iron-sulfur nanocluster. In fact, in biology, iron–sulfur clusters are metal cofactors that comprise the largest class of metalloproteins and are utilized for a wide variety of functions ranging from electron transport to DNA repair^9^.

The fact that modern-day enzyme biochemistry incorporates pyrite nanoclusters as active centres, points to a possible origin of pyrite on the prebiotic nitrogen fixation and production of ammonium^10,11^. However, previous ultra-high vacuum experiments to investigate the capability of pyrite to fix nitrogen, have been discouraging as they found that N_2_ does not dissociate on FeS_2_{100} under conditions where it would dissociate on Fe surfaces^12^. Interestingly, it has also been shown that a solution of FeS with 50–400 mg per mL of water can reduce atmospheric NO to ammonia^13^ in 2 to 3 hours of reaction time. Furthermore, materials with enhanced performance in terms of both adsorption capacity and strength of retention of ammonia are still needed, and it has been described that sulfur plays a role as sulfate ions can react with ammonia to form ammonium sulfate^14^. It is the purpose of this work to investigate the role of pyrite (iron-sulphur) on the fixation of molecular nitrogen on terrestrial planets like Mars and the Earth. Iron pyrite (FeS_2_) is the most abundant  sulfide mineral in the Earth’s crust. It has also been reported to exist on Mars: pyrite has been found by Curiosity at Gale crater^15^ and has been detected in the Allan Hills (ALH) 84001^16^ and in NWA7533 Martian meteorites^17^. Both meteorites preserve valuable information about the ancient Martian crust (∼4.5 Gyr)^18–20^. In 1977 Schrauzer and Guth demonstrated the photocatalytic reduction of atmospheric nitrogen to ammonia upon irradiation of rutile-TiO_2_ doped with 0.2% Fe_2_O_3_^21^. However, it was later on indicated that any NH_3_ produced with this process would be rapidly destroyed by photolysis and reactions with OH radicals^22^. The input of photons in semiconductors materials is indeed particularly interesting, because UV or VIS irradiation can promote the electrons from the valence band to the conduction band. This process creates the so-called electron-hole pairs that can easily react with adsorbed molecules on the surface of the semiconductor and trigger redox reactions. It has been shown that the photocatalytic activity of TiO_2_ can convert N_2_ and H_2_O to NH_3_^23,24^. This mechanism has been explained by the presence of oxygen vacancies on the TiO_2_ surface. Taking these cases as examples, in this work, we will investigate the enhanced catalytic properties of pyrite under exposure to UV radiation.

We postulate that the UV-photocatalytic interaction of stellar UV irradiance with mineral substrates may be effective mediators that enhance the binding of nitrogen in more reactive and life-friendly forms on terrestrial planets like Mars, Earth and other exoplanets. In this work, we describe a set of experimental laboratory studies that investigate the relationships and possible chemical reactions between different atmospheric conditions, N_2_, UV irradiation, and the catalysing influence of minerals surfaces like pyrite with respect to the fixation of nitrogen. The photocatalytic effects of pyrite on planetary atmospheres have to date been overlooked. The implications for the fixation of nitrogen on Earth and other terrestrial planets like Mars, are here investigated. The present-day atmosphere of Mars, is rich in CO_2_, which absorbs solar UV radiation for wavelengths below 200 nm. Whereas the present-day atmosphere of Earth, has ozone, which absorbs solar UV radiation in the UVC range, below 280 nm. Therefore, the UV radiation that reaches the surface of the Earth’s falls within the spectral range 280 nm–400 nm. For past atmospheric conditions, previous to the production of oxygen and then ozone by life, the UV reaching the surface of Earth must have reached wavelengths of 200 nm as in the case of Mars^25^. Our experiments will thus consider exposure to UV irradiance within the range 200 to 400 nm  to cover both cases and other terrestrial planetary environments with similar atmospheric conditions.

## Results and Discussion

### Pyrite surface UV-irradiated under high vacuum (HV) and air conditions

Previous studies have confirmed that iron and sulfur from pyrite surface are highly sensitive to chemical changes due to UV irradiation, even after very short times of 2 or 5 hours of UV exposition it has been confirmed that there exists an increase of iron oxidized species and the appearance of new oxidized and sulfates species^26^. A necessary condition for a potential effective catalyst is that it is easily affected by the environmental conditions. Indeed, pyrite is highly reactive and it can easily get oxidized, and thus we consider it as a candidate for our studies.

**Figure 1:**
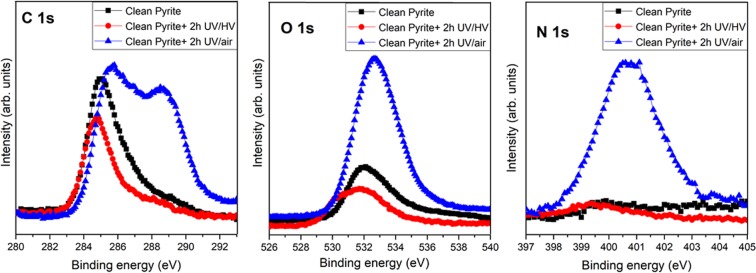
XPS photoemission spectra of C 1 s, N 1 s and O 1 s core level peaks of clean pyrite surface (black line), after 2 hours of UV irradiation under HV conditions (red line) and after 2 hours of UV irradiation at air conditions (blue line).

In order to fully characterize the pyrite surfaces chemistry under UV exposure, the carbon, oxygen and nitrogen signals have been measured by XPS (see Fig. 1). Clean pyrite (black reference line in Fig. 1) shows the characteristic feature from *ex-situ* samples, meaning samples manipulated at air conditions that are then transferred to UHV condition to measure the XPS signal. These samples show the presence of a carbon (at 285.0 eV) and oxygen (at 532.0 eV) component which can be clearly assigned to air contribution. No signal of nitrogen is observed. When the pyrite surface is exposed to UV radiation during two hours under high vacuum conditions (1 × 10^−5^ mbar), minor changes are observed in these peaks. These changes are due to the appearance of oxides species on the surface, and are characterised by a shift to lower binding energies both in the oxygen peak and the carbon peak (red spectrum in Fig. 1). Under HV conditions, there is still no signature of nitrogen. Thus, the C, O and N spectrum are very similar after UV exposure to the one of pristine, unperturbed, clean pyrite surface.

On the contrary, when the clean pyrite surface was exposed to UV irradiation at ambient air conditions (blue spectrum in Fig. 1) the spectrum dramatically changed. It is remarkable the increased intensity of oxides species (530–531 eV component) in the oxygen region, which are assigned to iron oxides, the appearance of a new carbon species at 288.5–290 eV, which is assigned to carbonates species and the appearance of a second component in the range of 286.5–287.5 eV that is assigned to C-N and C-S species. Finally, of particular relevance is the appearance of a strong nitrogen signal after UV irradiation at air conditions. This indicates that pyrite is able to fix nitrogen on its surface after 2 hours of exposure to a UV flux of F = 2270 mW m^−2^r within the range 200–400 nm in ambient atmospheric conditions.

**Figure 2:**
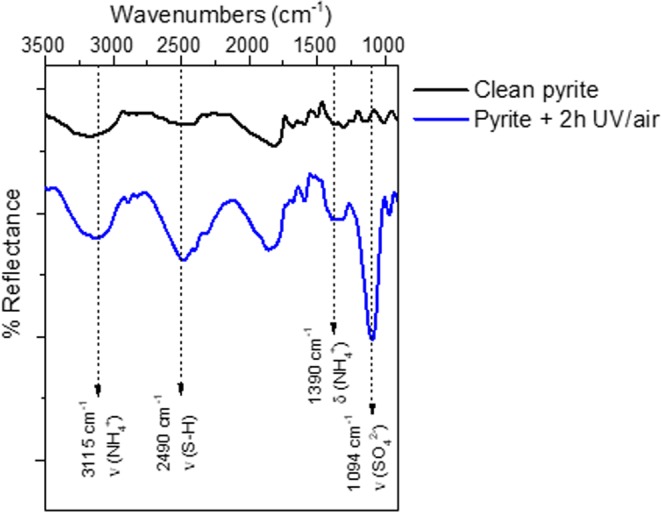
Infrared spectrum of clean pyrite surface (black line), after 2 hours of UV irradiation at air conditions (blue line). The arrows in the plot highlight the appearance of new vibrations frequencies after UV irradiation and the assignment of these bands^44,45^.

In order to further understand the chemical species involved in the nitrogen fixation process, we applied a detailed XPS analysis. The thorough components deconvolution of the UV oxidised pyrite surface reveals the presence of carbon, oxygen and nitrogen on the pyrite surface. The best-fit curve for the C 1s peak was obtained using three components. The first carbon component has a binding energy of 284.7 eV asigned to the air contribution and CH species, a second component at 286.4 eV and is attributed to the C-N and C-S groups^27,28^, whereas the third component is observed at 288.6 eV and is assigned to the carbonates groups. For the O 1 s, the peak is observed at 530.1 eV and is attributed to oxides species component, and a second component at 531.9 eV is assigned to the air contribution, sulfates (532.5 eV) and carbonates (531.5 eV) species, the third contribution at 533.6 eV (adsorbed H_2_O)^29^ is in the range of typical of carbonate type moities^30^, which also supports the observation of carbonate species in accordance with carbon (1s) signals. The best-fit curve of the N 1s peak consists of one component centred at binding energies of 400.9 eV, which could be assigned to the ammonium salts^31^. In order to confirm the appearance and accurate assignment of these new species, infrared analysis was performed (see IR spectrum and wavenumbers assignment, Fig. 2). Both complementary spectroscopies, XPS and infrared, help us to identify the nitrogen signal suggesting ferrous ammonium sulfate as the chemical species formed during the UV-photocatalytic nitrogen fixation process.

The exposure of pyrite surfaces to UV irradiation during two hours under high vacuum (HV) condition or at air condition shows similar features for the iron and sulfur spectra than clean pyrite under UHV conditions (not shown). However, for ambient conditions the spectrum changes dramatically for C and N (Fig. 1). The nitrogen feature appears only if the UV irradiation process take place in the presence of air. Under HV conditions, i.e. in the absence of a gaseous atmosphere above the surface, there is no nitrogen fixation on the surface. This control case demonstrates that the source of nitrogen is in the atmosphere. Furthermore, the carbon region shows the appearance of two new components, the first one due to the presence of C-N and C-S bonds at 286–287 eV and the second at 288.9 eV which is assigned to carbonates compounds^32^. These two signatures confirm that in the presence of air, the UV irradiation of pyrite induce the presence of carbonates species and nitrogen as amonium salt species on the pyrite surface.

We calculated the S/Fe ratio for different samples, in order to verify the FeS_2_ stoichiometry before and after UV irradiation under different environmental conditions. Clean pyrite (control) and UV irradiated pyrite under HV conditions show stoichiometric values between 2.1 and 2.2, which are in good agreement with the theoretical S/Fe value of 2. On the contrary, for UV irradiated pyrite at air conditions, the S/Fe ratio increases until 3.2, which points to a diminution of the iron signal on the surface after UV irradiation due to the adsorption of nitrogen and carbonates species. This suggests that nitrogen and carbonates species have a preference adsorption on the Fe sites. Of course, the pyrite composition ratio S/Fe does not change. Instead, the XPS signal from the surface-Fe atoms is attenuated as the nitrogen-species and carbonates molecules are adsorbing preferentially at the Fe sites, covering the pyrite Fe which is left underneath and thus diminishing the overall detectable signature of Fe, when compared to the one of sulfur.

### Role of the enviromental conditions on the efficiency of the nitrogen fixation

#### Increase of nitrogen fixation with atmospheric pressure

In order to deeply characterize the nitrogen fixation process on pyrite surface, and to understand how critical could be the exposure at diverse air pressure conditions, we have performed several experiments at different air pressure values of 1 × 10^3^ mbar and 7 mbar.

**Figure 3:**
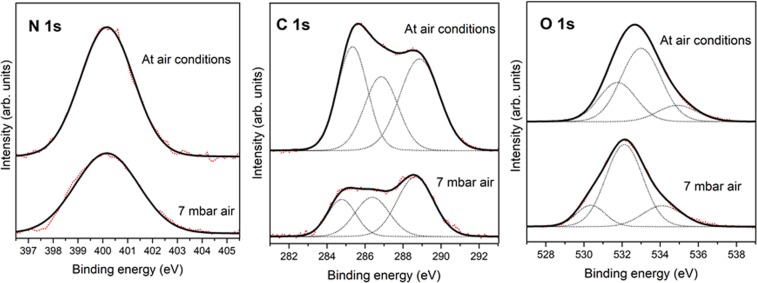
XPS photoemission spectra of N 1 s, C 1 s and O 1 s core level peaks of pyrite surface exposed to UV irradiation during two hours at air pressure values of 1 × 10^3^ mbar and 7 mbar air conditions.

The fixation process is taking place under both conditions (see Fig. 3, nitrogen region), confirmed by XPS, a nitrogen feature in the N(1s) spectra region. However, the nitrogen peak is more intense in the case of higher value of air pressure (1 × 10^3^ mbar). This is understandable as the molecular atmospheric density, and thus the nitrogen availability, is higher in the case of higher pressures.

On the other hand, the carbon region shows the same three components, which would be expected: at 284.5 eV, at 286.2 eV and at 288.6 eV. It is remarkable that carbonates species are dominant for 7 mbar air conditions instead of at 1 × 10^3^ mbar pressure value.

In conclusion, the air atmospheric pressure value is a significant parameter to take in account in the effectiveness and stages of the fixation nitrogen process on pyrite surface.

### Catalysis versus UV photo-catalysis at air conditions (1 × 10^3^ mbar)

A dedicated set of experiments have been done in the absence of the UV irradiation to evaluate the catalytic properties of pyrite alone. After the first two hours of ambient exposure there is no signature of nitrogen fixation on its surface (not shown).

**Figure 4:**
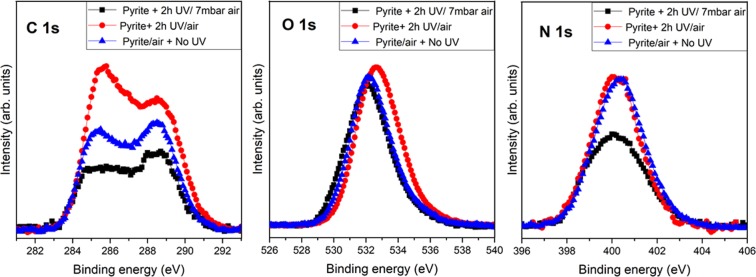
XPS photoemission spectra of C 1 s, N 1 s and O 1 s core level peaks of pyrite surface, after 2 hours of UV irradiation at 7 mbar air conditions (black line), after 2 hours of UV irradiation at air conditions (red line), and for long-term air exposure without UV radiation (blue line).

**Figure 5:**
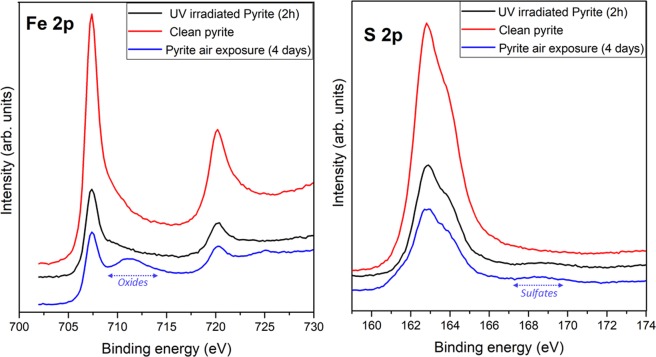
XPS photoemission spectra of Fe 2p and S 2p core level peaks of pyrite surface, for clean pyrite (red line), after 2 hours of UV irradiation at air conditions (black line) and for long-term air exposure (blue line).

We have investigated the long-term catalytic properties of pyrite in the absence of UV irradiation for a clean pyrite surface. Surprisingly, if we let the clean pyrite sample exposed to air conditions (1 × 10^3^ mbar) during several days (4–6 days), the air exposure alone generates oxides species and both nitrogen and carbonates signals are present on the pyrite surface (see Fig. 4, blue spectra). Even in the absence of UV irradiation, pyrite shows nitrogen fixation capabilities, however this catalytic property requires the presence of oxides species and longer time for the reaction which then takes place within 4–6 days (see Fig. 5, blue spectra, Fe region 708–713 eV, the oxides species region^33^). It is worth clarifying that N_2_ can be fixed nowadays in the absence of UV, however only after long-time exposure to present-day terrestrial oxygen-rich conditions, which allow the formation of oxides that seem to be the precursors for N_2_ transformation and fixation. Our experiments show that this catalytic process exists on pyrite also in the absence of UV (under present-day terrestrial oxygen-rich conditions), although 50 times slower, confirming the high enhanced efficiency of UV photo-catalysis on pyrite surfaces.

The nitrogen fixation process does not take place on a clean pyrite surface (absence of oxides species) without being UV irradiated. In the absence of UV, the presence of oxides species due to the pyrite air oxidation during 4 days (see oxides region for the blue spectrum of Fig. 5) are an essential factor to fix nitrogen on the pyrite surface.

Figure 5 shows a comparison of Fe 2p and S 2p spectrum, for clean pyrite surface no presence of oxides and sulfates species are detected (red spectrum), after UV irradiation of 2 hours (black spectrum) small peak from oxides species are detected whereas after 4 days of long-term exposure to air conditions (blue spectrum), it is remarkable the appearance of oxides and sulfates species which are necessary for perform nitrogen fixation process on pyrite surface in the absence of UV irradiation.

### Release of fixed nitrogen

**Figure 6:**
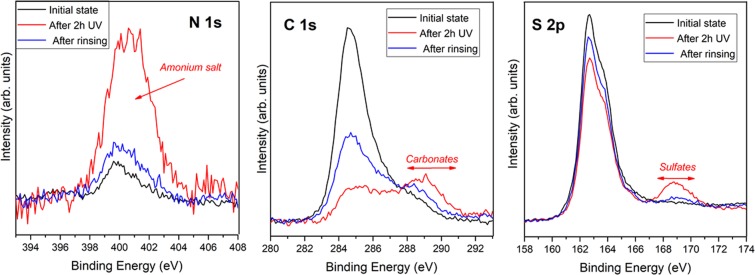
XPS photoemission spectra of N 1 s, C1s and S 2p core level peaks of pyrite surface, for clean pyrite (black line), after 2 hours of UV irradiation at air conditions (red line) and after water rinsing (blue line).

Our experiments have shown that UV photo-catalysis on pyrite can lead within a few hours to fixation of atmospheric N_2_ and water, in the form of an ammonium sulfate salt. Ammonium sulfate is an inorganic salt (which is actually commonly used as soil fertilizer) with a high solubility which easily disassociates into ammonium (NH_4_^+^) and sulfate (SO_4_^2−^) in aqueous solutions. Once the signature of nitrogen has been observed fixed on the surface of pyrite, we have rinsed the pyrite surface with 10 ml of milli Q water (pH of 5–6) at ambient conditions and repeated the XPS measurement. The analysis of XPS confirms the diminution of the nitrogen feature, and the decrease of the sulfates and carbonates species (see Fig. 6). This indicates that the nitrogen species are highly soluble in water, as expected for an ammonium salt, and is easily removed upon contact with liquid water. This implies, that a surface of pyrite exposed to a wet (liquid water) -dry (Solar irradiance) cycle would naturally fix within a few hours of solar exposure atmospheric nitrogen as ammonium and then release it when in contact with water. This ammonium is then available for prebiotic chemistry and life, while the pyrite surface is again exposed to the atmosphere and ready for a new catalytic reaction.

## Conclusions

We have demonstrated that pyrite catalyses the fixation of atmospheric nitrogen, the efficiency of this process increases with UV irradiation and atmospheric molecular density. XPS and IR spectroscopies have been used to identify independently the presence of ammonium salts on the surface of pyrite. This is furthermore confirmed by the easy release from the surface of the formed product with water. This kind of experiments can be used in the future to identify the most favourable conditions (in terms of temperature, atmospheric composition, absence or presence of liquid phases, UV ranges, rates of production etc.) to enhance the nitrogen fixation processes, to investigate other prebiotic or planetary environments and to investigate the possible induction of other reactions and the production of other volatiles (such as NOx…) that may act as intermediate factors and affect the atmospheric thermal behaviour. Additionally, this process may be of interest for the industrial production of ammonia.

Pyrite, FeS_2_, is a semiconductor with high iron content, is expected to be a strong UV absorber. The input of photons in semiconductors materials promote the electrons from the valence band to the conduction band. This process creates the so-called electron-hole pairs that can easily react with adsorbed molecules on the surface of the semiconductor and trigger redox reactions. Our work demonstrates that pyrite has UV-photocatalytic activity, and even catalytic activity (in the absence of UV) provided that oxides species are present on the surface. The presence of oxides and carbonates species, detected simultaneously by XPS when the nitrogen signal appears, suggests that they might play a crucial role as intermediate species in the process, because in their absence the adsorption phenomenon is not observed. Further experiments are underway to elucidate their role. The appearance of the nitrogen signal is always linked to the presence of carbonates and oxides species. Oxidised surface species come from UV irradiation or surface air exposure for several days. Previous works have assessed the enhancement of the photocatalytic activity of pyrite in presence of oxides^34,35^. Furthermore Evangelou *et al*. hypothesized that pyrite surface-CO_2_ complexes could promote abiotic oxidation of pyrite by accelerating the abiotic oxidation of Fe^2+^. The data show that the presence of NaHCO_3_ significantly increased the oxidation rate of FeS_2_^36^.

Our experimental results showed that the UV-irradiated pyrite surface is able to fix N_2_ and form NH_4_^+^ in a few hours. We hypothesized that the sulfur vacancies on FeS_2_ surface destabilize the neighbour iron^26,37^ allowing to cleavage the N_2_ molecule and transforming to NH_4_^+^ in the presence of the adsorbed H_2_O. Indeed, previous works have proved that these defect sites are capable of forming reactive oxygen species (ROS) by water splitting under anaerobic conditions^11,38^. Furthermore, it has been described that ammonia reactive adsorption in the presence of oxygen-containing groups leads to the formation of ammonium ions, then in the presence of sulfur compounds formation of ammonium sulfates is observed via oxidation of sulfonic groups by active oxygen^14^, and these salts are stable for example on carbon surfaces at ambient conditions.

We have reported that solar levels of UV radiation can fix atmospheric nitrogen within a few hours provided that pyrite acts as a catalyst. This process leads therefore to nitrogen sequestration and may have been active in the prebiotic era on Earth, as it may be active on other terrestrial planets with UV transparent atmospheres and catalytic minerals reducing the levels of nitrogen in the atmosphere and thus having an impact on the radiative balance of the planet. This process has furthermore implication for the abiotic nitrogen fixation on other planetary environments, and it has critical implications for the habitability of planet and the origin of life. Future studies will focus on investigating the role of different atmospheric compositions (varying the concentration of N_2_, CO_2_, H_2_O etc) and pressures, to characterize the efficiency of this process for different plausible planetary atmospheres. The experimental set-up and tests are in progress. We conclude that UV photocatalysis on pyrite may have been a natural mechanism of prebiotic fixation of nitrogen into ammonium sulfates which is then easily released upon contact with liquid water. This property of pyrite may have been incorporated naturally in the prebiotic chemistry evolution, leading to the inclusion of pyrite nano-clusters as reaction centres to generate ammonia from nitrogen, and then from ammonia to generate ammonium sulfates salts in the presence of oxygen. Furthermore, photocatalytic N_2_ fixation is relevant in the interdisciplinary fields of chemistry, materials science, energy conversion and energy storage^39^.

## Materials and Methods

**Figure 7:**
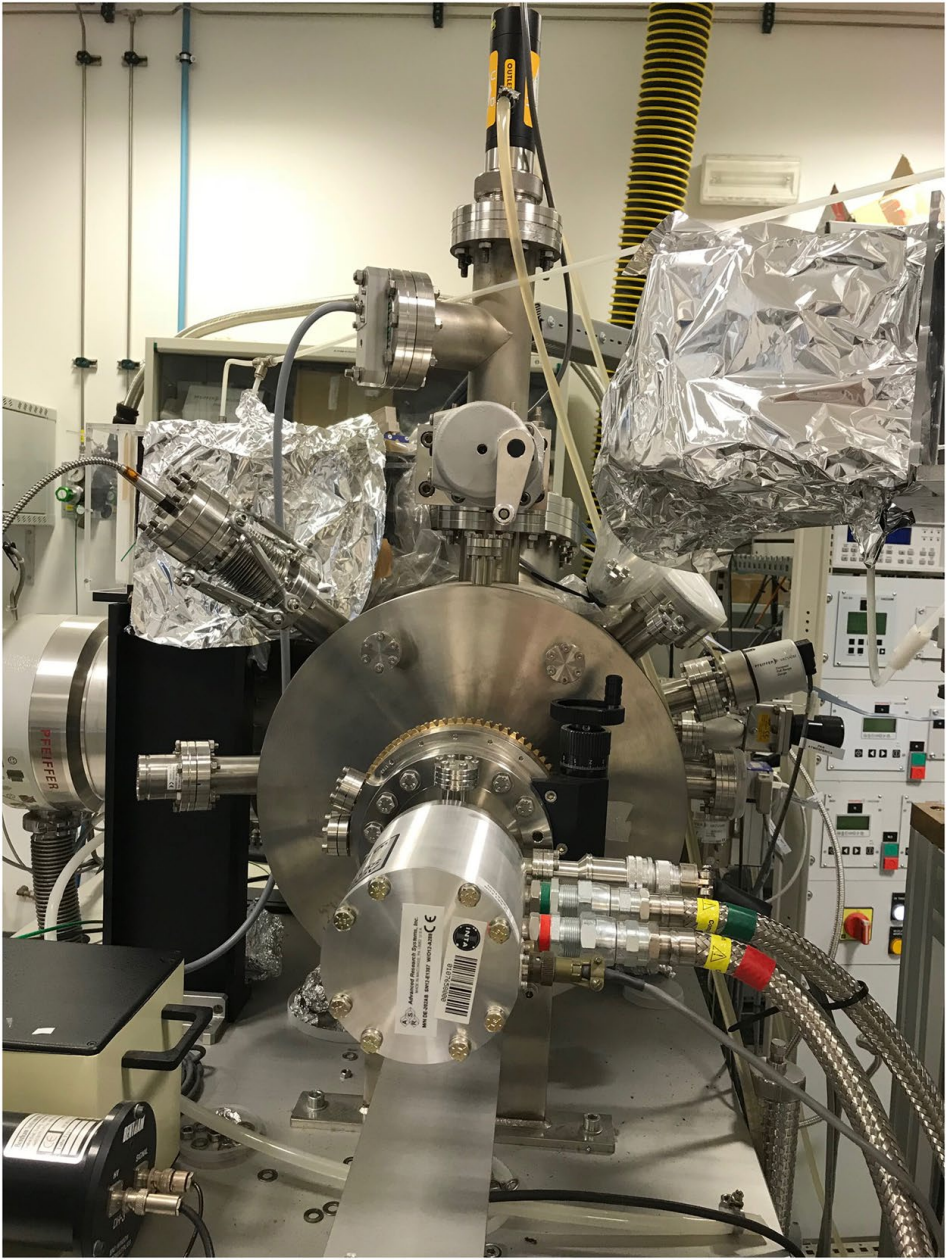
*Planetary Atmosphere and Surfaces Chamber (PASC)* is an ultra-high-vacuum (UHV) simulation chamber 500 mm long by 400 mm diameter that is capable of reproducing atmospheric compositions and surface temperatures that are representative of most planetary objects. This equipment was specifically developed to make feasible the *in situ* UV irradiation (200–400 nm) of samples under study. The total pressure range of the chamber is from 5 mbar to 5 × 10^−9^ mbar.

A sample of pyrite (from Navajun mine Spain) was cleaned three times in different solutions of 1 M H_2_SO_4_, immersed in water (milli-Q grade) and then dried by blowing compressed air. Then, the pyrite sample was transferred to an ultra-high vacuum (UHV) chamber with a base pressure of 3 × 10^−10^ mbar, for the X-ray Photoelectron Spectroscopy (XPS) measurements. In the cases where it was required to store the pyrite samples overnight prior to running the experiment the following day, the sample was kept under vacuum conditions to avoid sample degradation or contamination. The experimental set-up for the UV irradiation experiments was performed inside the planetary atmospheres and surfaces chamber (PASC), a dedicated planetary simulation chamber (see Fig. 7 and technical details Mateo-Martí *et al*.^40)^. Then, pyrite samples were transferred to a high vacuum (HV) pre-chamber with a base pressure of 3 × 10^−7^ mbar, and then transferred inside of the UHV condition chamber for the X-ray Photoelectron Spectroscopy (XPS) measurements. Same experiments have been repeated several times and in different pyrite samples in order to confirm the reproducibility of the nitrogen fixation process. Several experiments have been carried out with pyrite under different atmospheric conditions (see Table 1) to confirm which experimental conditions favour the N_2_ fixation process.

**Table 1:** Summary of experiments carried out with pyrite under different atmospheric conditions.

UV	UHV	7 mbar Air	Air
ON	Pyrite/No successful N_2_	Pyrite (2 h)/Yes successful N_2_	Pyrite (2 h)/Yes successful N_2_
OFF			Pyrite (4 days)/Yes successful N_2_

### UV irradiation

A 150 W water-cooled deuterium UV lamp (Hamamatsu C3150), placed perpendicular to the pyrite sample, was used to irradiate the sample. The clean pyrite surface was exposed to UV radiation (200–400 nm) for 2 hours. The UV radiation from the lamp enters the system through a quartz window. The UV light hits a beam splitter, placed very close to the lamp, which allows 88% of the radiation to pass through. The other 12% of the beam is reflected onto another quartz window, where a UV detector is placed that permits the continuous monitoring of the incoming UV flux via a spectrum-radiometer (Bentham DMc150FC). After the beam splitter, we set a focusing lens to focus the beam on the surface. The irradiance spectrum of the deuterium lamp is a continuum that decreases for increasing photon wavelength. The UV flux measured at the sample position, can be obtained by integration of the irradiance measured by the radiometer over the 200–400 nm wavelength range. The resulting flux is F = 2270 mW m^−2^, which corresponds to F = 2.3 10^14^ (6 eV photons) cm^−2^ s^−1^^41^. This can be considered a natural solar UV exposure for a primordial Martian or Terrestrial atmosphere, namely it is about 10 times smaller than the UV 200–400 nm flux at equatorial noon on the Martian surface, and 20 times smaller than the UV 200–400 nm flux on the Earth surface^25,42,43^.

The UV irradiation was performed for periods of 1 to 2 hours with ambient air earth conditions (temperature of 22 °C and 45% of humidity), in two pressures ranges of: (1) Earth ambient conditions (1 × 10^3^ mbar); and (2) Mars conditions (7 mbar). For comparison, it was also performed at high vacuum conditions (1 × 10^–6^ mbar), i.e. in the absence of an atmosphere.

### Long term ambient exposure

Pyrite samples were cleaned three times in different solutions of 1 M H_2_SO_4_, immersed in water (milli-Q grade) and then dried by blowing compressed air. The clean pyrite samples were exposed to air earth conditions (1 × 10^3^ mbar, standard earth atmosphere composition) during several days (4–6 days) in a laboratory bench. Samples were placed inside of a plastic container to avoid dust deposition on the surface.

**Figure 8:**
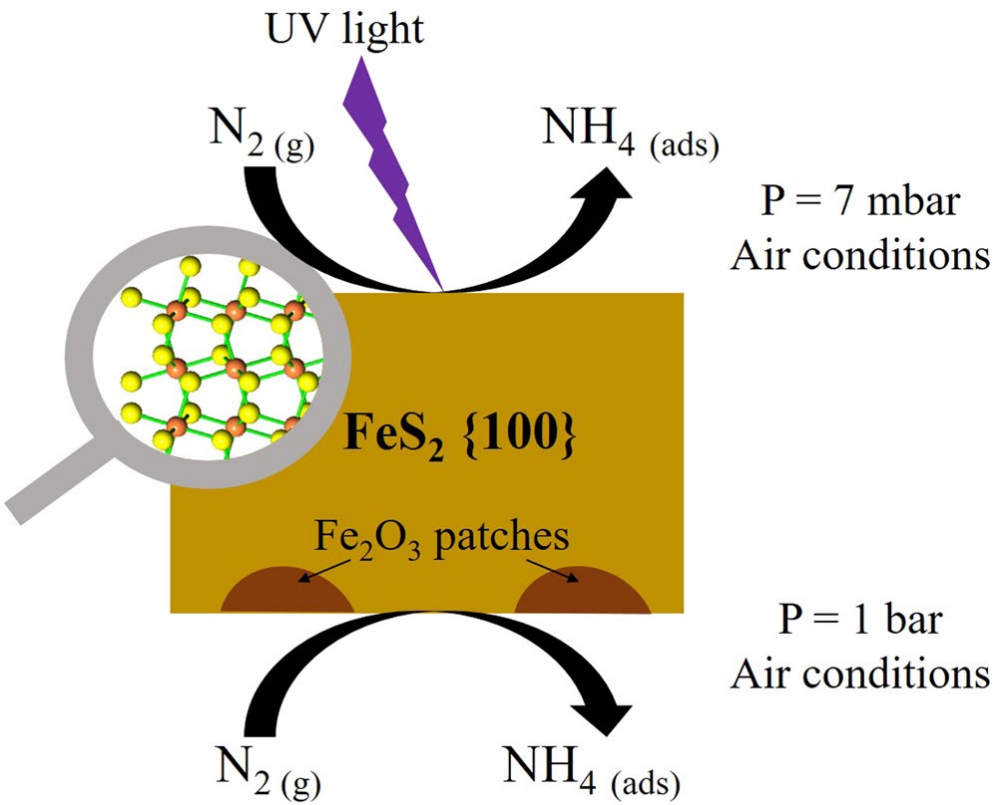
Schematic representation of the processes that lead nitrogen fixation on pyrite surface, (i) by UV photo-catalysis under low pressure conditions (on the top) and, (ii) by the catalytic effect of iron oxide-iron sulfide tandem under visible light conditions and standard earth atmosphere (on the bottom).

Figure 8 shows a schematic representation of the processes that lead nitrogen fixation on pyrite surface: by UV photo-catalysis (low pressure conditions) and by the catalytic effect of iron oxide-iron sulfide tandem under visible light conditions and standard earth atmosphere.

### XPS analysis

The XPS spectra of the sample were recorded after the exposure to UV radiation in a different dedicated XPS chamber. The XPS of the pristine clean pyrite surface was also recorded in order to get information about the surface before UV irradiation. The XPS analysis of the samples was carried out in an ultra-high vacuum chamber equipped with a hemispherical electron analyzer and with the use of an Al Kα X-ray source (1486.6 eV) with an aperture of 7 mm × 20 mm. The base pressure in the chamber was 5 × 10^−10^ mbar, and the experiments were performed at room temperature. The peak decomposition in different components was shaped, after background subtraction, as a convolution of Lorenztian and Gaussian curves. Binding energies were calibrated against the binding energy of the Fe 2p_3/2_ peak at 707.3 eV for the pyrite samples. We have not observed any beam radiation damage of the pyrite surface during the data acquisition.

### IR analysis

Fourier-transform infrared (FTIR) spectroscopy of the pyrite samples was performed in a thermo-Nicolet spectrometer. Spectra (2 cm^−1^ of resolution and 128 scans) were collected in the mid-infrared region (400–4000 cm^−1^), using a DTGS-ATR detector and a XT-KBr beamsplitter.
